# Geography as non-genetic modulation factor of chicken cecal microbiota

**DOI:** 10.1371/journal.pone.0244724

**Published:** 2021-01-06

**Authors:** Natalia Pin Viso, Enzo Redondo, Juan María Díaz Carrasco, Leandro Redondo, Julia Sabio y. Garcia, Mariano Fernández Miyakawa, Marisa Diana Farber

**Affiliations:** 1 Instituto de Agrobiotecnología y Biología Molecular, IABiMo, INTA-CONICET, Calle Las Cabañas y Los Reseros s/n, Castelar, Buenos Aires, Argentina; 2 Consejo Nacional de Investigaciones Científicas y Técnicas, Ciudad Autónoma de Buenos Aires, Buenos Aires, Argentina; 3 Instituto de Patobiología Veterinaria, IPVet, INTA-CONICET, Calle Las Cabañas y Los Reseros s/n, Castelar, Buenos Aires, Argentina; Universidad Nacional Autonoma de Mexico, MEXICO

## Abstract

The gastrointestinal tract of chickens harbors a highly diverse microbiota contributing not only to nutrition, but also to the physiological development of the gastrointestinal tract. Microbiota composition depends on many factors such as the portion of the intestine as well as the diet, age, genotype, or geographical origin of birds. The aim of the present study was to demonstrate the influence of the geographical location over the cecal microbiota from broilers. We used metabarcoding sequencing datasets of the 16S rRNA gene publicly available to compare the composition of the Argentine microbiota against the microbiota of broilers from another seven countries (Germany, Australia, Croatia, Slovenia, United States of America, Hungary, and Malaysia). Geographical location played a dominant role in shaping chicken gut microbiota (Adonis R2 = 0.6325, *P* = 0.001; Mantel statistic r = 0.1524, *P* = 4e-04) over any other evaluated factor. The geographical origin particularly affected the relative abundance of the families Bacteroidaceae, Lactobacillaceae, Lachnospiraceae, Ruminococcaceae, and Clostridiaceae. Because of the evident divergence of microbiota among countries we coined the term “local microbiota” as convergent feature that conflates non-genetic factors, in the perspective of human-environmental geography. Local microbiota should be taken into consideration as a native overall threshold value for further appraisals when testing the production performance and performing correlation analysis of gut microbiota modulation against different kind of diet and/or management approaches. In this regard, we described the Argentine poultry cecal microbiota by means of samples both from experimental trials and commercial farms. Likewise, we were able to identify a core microbiota composed of 65 operational taxonomic units assigned to seven phyla and 38 families, with the four most abundant taxa belonging to *Bacteroides* genus, Rikenellaceae family, Clostridiales order, and Ruminococcaceae family.

## Introduction

The chicken gastrointestinal tract (GIT) harbors a very diverse microbiota, dominated by Bacteria, that influences health and growth performance of chicken. A healthy microbiota provides nutrients to the host and promotes competitive exclusion [1]. The composition of the GIT microbiota differs according to diet, age and genotype of hosts as well as the portion of the intestinal tract, among others [2]). The poultry industry has adopted the use of dietary additives because of their anti-microbial and/or growth-promoting effects. To date, many additives are available, including antibiotics administered in sub-therapeutic doses, prebiotic, probiotics, organic acids and plant extracts [3]).

In the last few years, researchers have been paying closer attention to the influence of the geographical origin on microbiota composition. Indeed, in humans, geography can explain part of the observed variability on intestinal bacterial communities [4, 5]. In birds, environmental parameters seem to be the most important factors shaping host-associated microbiota [6, 7]. For example, fecal microbial composition of egg laying hens and poultry vary between samples from different geographical origins of Europe, such as Slovenia, Hungary, Croatia, and Czech Republic [8]. More recently, by comparing samples from five high altitude regions of China, ‬‬‬‬‬‬Zhou *et al*. [9] determined that the cecal composition of the intestinal microbiota of Tibetan chickens is altered by their origin.‬‬‬‬‬‬‬‬‬‬‬‬‬‬‬‬‬‬‬‬‬‬‬‬‬‬‬‬‬‬‬‬‬‬‬‬‬‬‬‬‬

With all this in mind, we hypothesized that the geographical location is a key modulator factor of cecal microbiota. In this regard, we considered geography in terms of the “human-environment” theoretical framework [10], overcoming the physical and a human geography divide. Location, as an idealized geographical space [11], would explain flows and interactions between factors like climate, farm management (including health interventions, nutrition, feed and litter management), and socioeconomic and cultural setting. To test this hypothesis, we reanalyzed publicly available data from NCBI and MG-RAST from eight different countries and moved forward using local data for describing the Argentine chicken cecal microbiota.

## Materials and methods

### Data acquisition from public databases

To compare the composition of chicken’s GIT microbiota from different geographic locations, short amplicon data from next-generation sequencing experimental trials (ET) were used. Datasets were obtained from MG-RAST and the NCBI Sequence Read Archive. Data sources, corresponding to eight different countries, including Argentina, are reported in Table 1. All downloaded data were re-analyzed using all samples as one large data set, using Quantitative Insights Into Microbial Ecology (QIIME) v1.9.1 software [12].

**Table 1:** 16S rRNA gene amplicon data used to compare the chicken GIT microbial composition throughout geographic location.

Source/ID	Geographic location	Intestinal portion	Age (days)	Genetic line	Region sequenced	Diet	Extraction kit	Platform	Reference
**NCBI**									
PRJEB9198	GER	Cecum	25	Ross	complete	SCD, MCP	Qiagen	Roche	[13]
SAMN03092832-39	MAL	Ileum/Cecum	21/42	Cobb	V3	SCD	Qiagen	Illumina	[14]
SRP045877	CRO	Fecal	21	Ross/Cobb	V3-V4	SCD	Qiagen	Roche	[8]
SRP045877	SLO	Fecal	21	Ross/Cobb	V3-V4	SCD	Qiagen	Roche	[8]
SRP045877	HUN	Fecal	21	Ross/Cobb	V3-V4	SCD	Qiagen	Roche	[8]
SAMN03161778-871	USA	Cecum	42	Ross/Cobb	V1-V3	SCD, OA	[15]	Roche	[16]
**MG-RAST**									
4614960.3	AUS	Cecum	25	Cobb	V1-V3	SCD	[17]	Roche	[18]
**AVAILABLE UNDER REQUEST**									
--	ARG-ET1	Cecum	26	Cobb	V3-V4	SCD, Bac, Tan	Qiagen	Illumina	[19]
--	ARG-ET2	Cecum	22	Cobb	V3-V4	SCD, Tan	Qiagen	Illumina	Díaz Carrasco Unpublished

GER: Germany, MAL: Malaysia, CRO: Croatia, SLO: Slovenia, HUN: Hungary, USA: United States, AUS: Australia, ARG-ET1: Argentina-Experimental Trial 1, ARG-ET2: Argentina-Experimental Trial 2. SCD: Standardized commercial diet for feeding broilers. MCP: monocalcium phosphate; OA (Organic acids): formic acid, propionic acid, ammonium formate and medium-chain fatty acids; Bac: subtherapeutic levels of zinc bacitracin; Tan: blend of tannins derived from chestnut and quebracho. Qiagen: QIAmp DNA Stool Mini Kit. Roche: Roche-454. Illumina: Illumina-MiSeq.

### Argentine cecal microbiota samples from commercial farms

We selected 10 commercial broiler farms from Argentina, classified according to husbandry practices into conventional poultry (CP) and agroecological farm (AE). Cecal content were collected from 27 samples in total, coming from 9 CP and 1 AE (S1 Table). To reduce inter individual variation each sample is the pool of the cecal content from five animals per pen, after cervical dislocation euthanizing proceeding. All cecum samples were immediately refrigerated on ice and then stored at −80°C until DNA extraction.

The animal experiments reported in this manuscript were conducted in accordance to protocol number 20/2010 from the Institutional Committee for the care and use of animals-INTA (CICUAE Approved by resolution CICVyA No. 14/07) based on internationally recognized guidelines of ‘‘Care and Use of Experimental Animals” as Guide for the Care and Use of Agricultural Animals in Research and Teaching, 3rd edition, 2010. Participation in the study was voluntary.

### DNA extraction and sequencing

Total genomic DNA was isolated from 300 mg of cecal content using QIAamp DNA Stool Mini Kit (Qiagen, Hilden, Germany) according to the manufacturer's recommendations. DNA concentration and purity were assessed in NanoDrop ND−1000 spectrophotometer (NanoDrop Technologies, DE, USA) and DNA was stored at −20°C until further analysis. The V3-V4 region of bacterial 16S rRNA gene amplification from the total extracted DNA, together with the construction of the 16S gene libraries and high−throughput sequencing using the Illumina MiSeq platform were performed at Macrogen Inc. (Seoul, South Korea). The generated paired-end reads of 300bp were obtained with primers b341F (5’-CCTACGGGNGGCWGCAG-3’) and Bakt805R (5’-GACTACHVGGGT ATCTAATCC-3’).

All sequence data were deposited in the NCBI Sequence Read Archive database under the BioProject accession number PRJNA579062.

### Microbial community analysis

Microbial community was analyzed by using QIIME v. 1.9.1 with default command parameters, unless specified. An average *phred* quality score threshold higher than 20 was used to filter low quality reads from raw sequence reads. Paired-end reads were joined and potentially chimeric sequences were identified and filtered using UCHIME algorithm [20]. All sequence data were then clustered into operational taxonomic units (OTUs) at 97% similarity against the GreenGenes database version 13.8, using UCLUST algorithm [21]. OTUs with abundance below 0.005% were filtered and the remaining OTUs were normalized using the total-sum scaling method (which divides the number of sequences per OTU by the total number of sequences in the sample).

For the geographical analysis, due to the broad range of 16S gene regions under analysis, we used the ‘‘closed reference” approach. This method discarded reads that failed to match the reference sequences, thus taxonomies came directly from the reference database upon the identity of the reference sequence clustered against.

Microbial diversity was evaluated within samples (alpha diversity) and between samples (beta diversity) using QIIME. Alpha diversity was assessed by richness (Chao1 index and observed OTUs) and community diversity (Shannon and Simpson indexes). The beta diversity in the microbial communities was evaluated on square root transformed OTU abundances; hierarchical clustering was performed on Bray-Curtis dissimilarity by using an average method to grouping the microbiotas in RStudio software with *vegan* package [22]. Finally, UniFrac analysis [23] and unweighted principal coordinate plots (PCoA) were used. For geographical analysis we used the 97% OTUs phylogenetic tree supplied with Greengenes. QIIME scripts used and the intermediate results for this analysis are available from figshare: https://doi.org/10.6084/m9.figshare.c.4993856.

### Statistical analysis of results

To compare the microbial composition of GIT samples from different countries, multiple rarefactions 100 times with averaging count where performing. The output file was further analyzed using Statistical Analysis of Metagenomic Profiles (STAMP) software [24], with ANOVA and Bonferroni correction to identify differentially OTUs abundances. Single sample per country was considered as the experimental unit. To find differences in the alpha diversity indexes, Kruskal-Wallis and Mann-Whitney post-hoc test was realized, corrected by Bonferroni method. For the beta diversity index, the grouping of samples based on each metadata factor, after the PCoA based on unweighted UniFrac distances, was evaluated using non-parametric multivariate ADONIS statistical analysis in QIIME. Additionally, Mantel test, wrapped in RStudio, was performed with 9999 permutations for appraisal the correlation between the unweighted UniFrac distances with geographical ones. We used https://www.geodatos.net/ for geographical coordinates and Haversine distances were calculated using *geosphere* package in R.

For all the statistical analysis, differences at *P* < 0.05 were considered significant.

## Results

### Geographic location shapes the cecal microbiota

**Fig 1:**
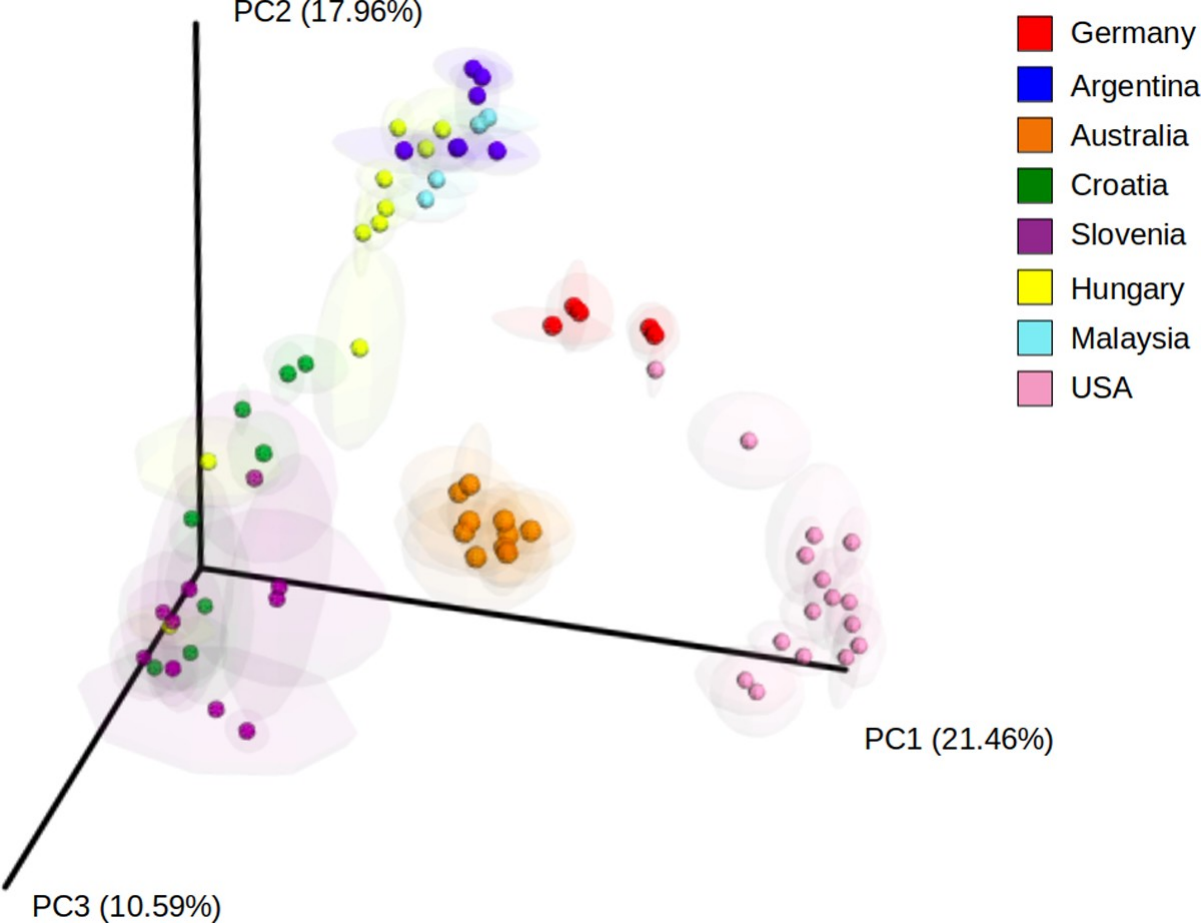
Principal coordinate analysis (PCoA) of unweighted UniFrac distances. Chicken GIT microbiota samples from different geographic locations were designated as AUS: Australia, ARG: Argentina, CRO: Croatia, GER: Germany, HUN: Hungary, MAL: Malaysia, SLO: Slovenia and USA: United States.

By means of comparison analysis of downloaded data from public repositories of eight different countries, we tested the influence of the geographical location on GIT microbiota. The arrangement that arises by the multidimensional scaling analysis (PCoA based on unweighted UniFrac distances of the 16S rRNA gene) revealed distinct groups (Fig 1). Additionally, the fit (63.25% observable variability) of the metadata factors (Table 2) revealed the geographic location as the strongest driver of community structure. On top of that, the Mantel test outcome showed statistically significant correlation between beta diversity and location distance matrices (Mantel statistic r = 0.1524; Significance = 4e-04).

**Table 2:** Calculated fit of metadata factors to unweighted UniFrac community distances for chicken microbiota using ADONIS.

Unweighted UniFrac	R2
Geographic location	0.63
Region sequenced	0.35
Diet	0.36
Extraction Kit	0.33
Intestinal portion	0.22
Age (days)	0.20
Genetic line	0.17
Platform	0.11

Statistical significance identified for all factor (*P* < 0.01).

**Fig 2:**
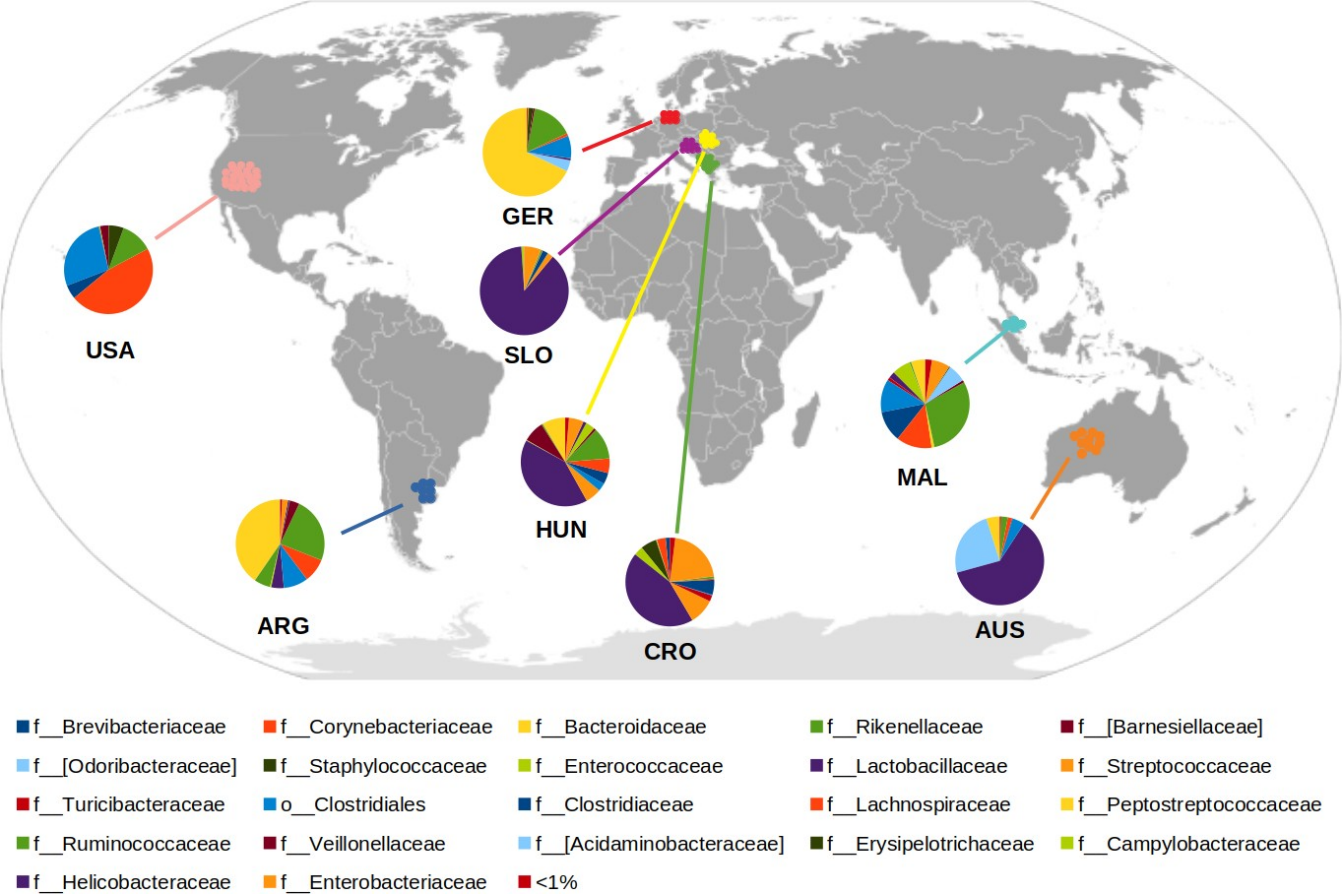
Geography map of chicken GIT microbiota at the family level. Different colors are used to indicate each individual taxon according to the country of origin designated as AUS: Australia, ARG: Argentina, CRO: Croatia, GER: Germany, HUN: Hungary, MAL: Malaysia, SLO: Slovenia, and USA: United States. The taxonomic classification: p_phylum, c_class, o_order, and f_family.

On the other hand, the use of Bray-Curtis dissimilarity metrics in a clustering analysis of microbial communities at the genus level yielded similar results (S1 Fig). Moreover, the relative OTU abundances at the family level also support the community structures associated with geography (Figs 2 and S2 and S2 Table). Notably, the presence of Bacteroidaceae, Lactobacillaceae, Lachnospiraceae, Ruminococcaceae, and Clostridiaceae explained the detected differences (S2 Table).

Additionally, we performed an analysis of the microbiota of each country regarding richness and evenness. In this regard, the community structure of Malaysian microbiota was the most diverse with the higher values of alpha diversity indexes. In contrast, the microbiota from Croatia and USA showed the lower values. Slovenia and Croatia showed no significant statistical differences across Chao1 and Shannon diversity index. These two countries grouped together according to both, PCoA distribution and alpha diversity indexes values. Similarly, alpha and beta values did not support the geographical distance between Hungary, Argentina, and Malaysia (Table 3).

**Table 3:** Alpha diversity indexes for chicken samples throughout geographic location.

Geographic location	OTUs per sample	Chao1 index	Shannon diversity	Simpson diversity
United States	31.39±5.76a	37.59±8.78a	3.09±0.85a	0.74±0.18ab
Croatia	42.10±26.50a	55.31±28.10ab	3.56±0.81ab	0.84±0.10cd
Germany	44.17±7.33ab	56.65±8.40abc	2.90±0.42a	0.69±0.10a
Slovenia	59.30±10.85bc	74.20±18.98bc	2.81±0.61a	0.72±0.13a
Australia	86.70±15.49bcd	95.61±20.93cd	3.25±0.20a	0.81±0.04abc
Hungary	124.70±43.23cd	169.41±58.55d	4.06±0.82bc	0.85±0.10bcd
Argentina	163.29±32.72d	189.99±43.41d	4.57±0.25c	0.90±0.01d
Malaysia	590.00±35.38e	621.28±27.78e	5.92±0.92c	0.94±0.04d

Mean±SD are showing. Different letters indicate significant differences among samples according to Kruskal Wallis with Mann-Whitney post-hoc test (*P* < 0.05) and Bonferroni correction.

### Characterization of Argentine cecal microbiota

We explored two different categories: chickens reared under experimental trial (ET) and commercial broiler farms (CF). CF included conventional poultry (CP) and one agroecological farm (AE). The analysis consisted of ET samples involves in the previously analysis, and other 17 samples of the same datasets from different ages (Table 1), and 27 samples obtained from CF (S1 Table). A total of 3032606 quality trimmed sequences and 1172 different OTUs, were obtained with an average number of sequences per sample of 49168, 49881, 69817, and 49297, for ET1, ET2, CP, and AE respectively.

**Fig 3:**
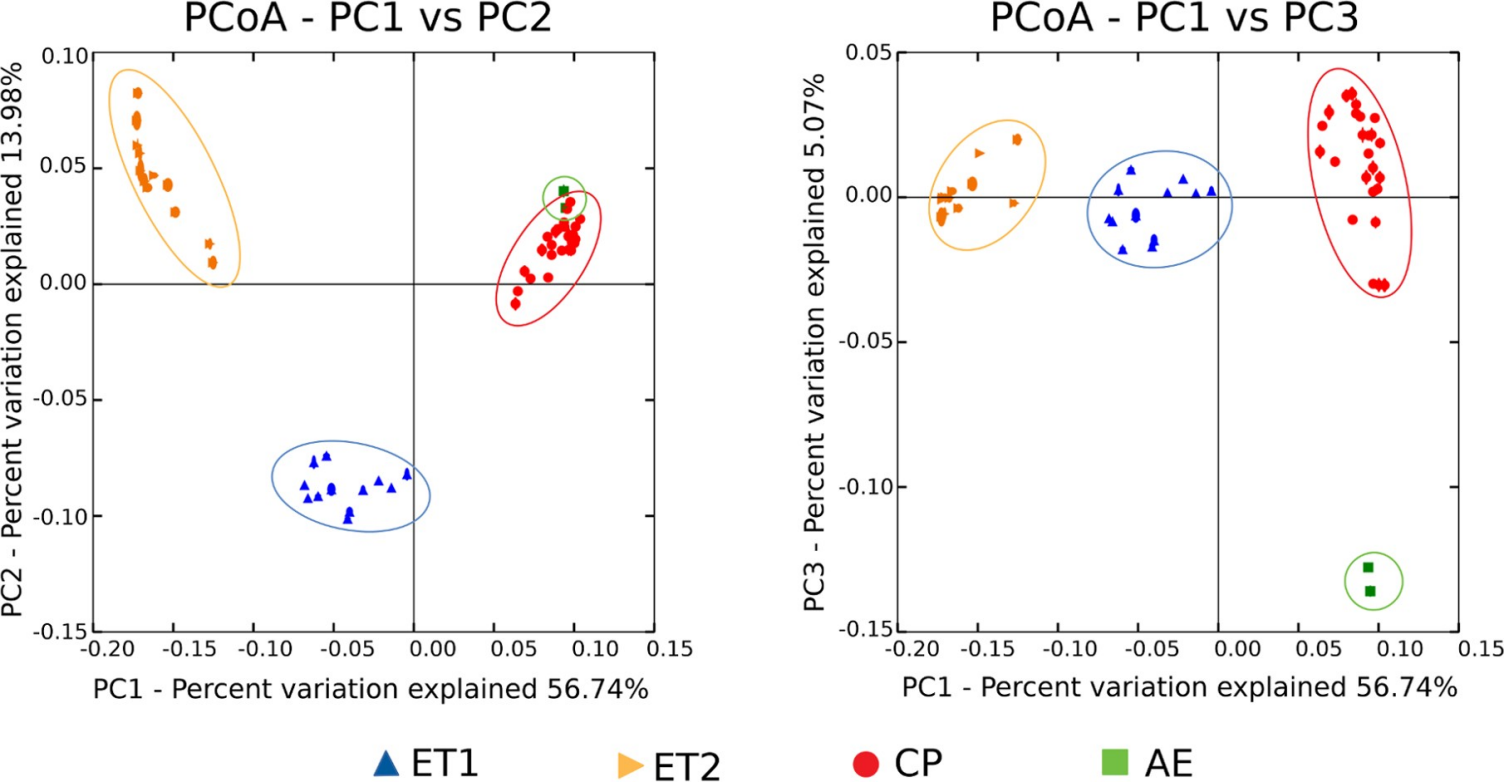
Principal coordinate analysis (PCoA) of unweighted UniFrac distances. Chicken cecal microbiota samples from different Argentinian farms were designated as ET1: Experimental Trial 1, ET2: Experimental Trial 2, CP: Conventional Poultry, and AE: Agroecological Farm.

The distribution of the experimental and commercial datasets gathered into different groups according to the multidimensional scaling analysis (PCoA based on unweighted UniFrac distances of the 16S rRNA gene). PC1 clearly showed the differences between experimental and commercial datasets, whereas PC2 displayed differences between each experimental trial (ET1 and ET2). Finally, PC3 allowed us to separate the data belonging to AE and CP (Fig 3). In addition, alpha diversity values confirmed two separate groups (ET and CF) according to both richness and diversity indexes (Table 4).

**Table 4:** Alpha diversity indexes for chicken cecal Argentine samples.

Sample	OTUs per sample	Chao1 index	Shannon diversity	Simpson diversity	Good's coverage
ET1	328.92±34.20 a	367.05±34.88 a	5.27±0.34 a	0.91±0.02 a	0.99±0.01
ET2	189.58±39.60 b	218.81±49.63 b	4.63±0.45 b	0.90±0.04 a	0.99±0.01
CP	571.04±37.11 c	623.73±43.90 c	6.56±0.29 c	0.97±0.01 b	0.99±0.01
AE	550.50±17.68 c	614.39±19.87 c	6.82±0.11 c	0.98±0.00 b	0.99±0.01

Mean±SD are showing. Different letters indicate significant differences among samples according to Kruskal Wallis with Mann-Whitney post-hoc test (*P* < 0.05) and Bonferroni correction. ET1: Experimental Trial 1, ET2: Experimental Trial 2, CP: Conventional Poultry, AE: Agroecological Farm

As expected for cecal microbiota, Firmicutes, Bacteroidetes, and Proteobacteria represented the major phyla regarding community structure. For CF, their relative abundances were 45.79%, 46.75%, and 2.52% for CP and of 27.11%, 38.36%, and 8.01% for AE, respectively. The unclassified bacteria ascended to 15.71% in AE samples and 2% in CP. Within the Firmicutes phylum, Clostridia was the dominant class (41.20% for CP and 25.7% for AE), with a prominence of the Clostridiales order. From this order, the prevalent families were Ruminococcaceae and Lachnospiraceae in CP (18.22% and 5.43% respectively) and three families, Ruminococcaceae (4.96%), Lachnospiraceae (4.35%), and Veillonelaceae (7.35%), displayed similar relative abundance in AE samples. Within the Bacteroidetes phylum, Bacteroidales of the Bacteroidea class was a highly abundant order (46.75% for CP and 38.35% for AE). The more representative families from Bacteroidales were Bacteroidaceae (18.56% and 18.28%), Rikenellaceae (15.47% and 1.46%), and Barnesiellaceae (8.39% and 1.27%).

On the other hand, the microbiota of ET was dominated by Firmicutes (50.67%), followed by Bacteroidetes (44.84%), and Proteobacteria (3.41%). Within the Firmicutes phylum, Clostridia was the dominant class (47.23%) among the most abundant members from the Clostridiales order. The most abundant families were Ruminococcaceae and Lachnospiraceae (17.64% and 7.82% respectively). Within the Bacteroidetes phylum, Bacteroidales was the most abundant order of the Bacteroidea class (44.84%). In this case, the most representative families were Bacteroidaceae (33.47%), Rikenellaceae (5.86%), and Barnesiellaceae (5.50%). S3 Table shows the statistical analyses.

**Fig 4:**
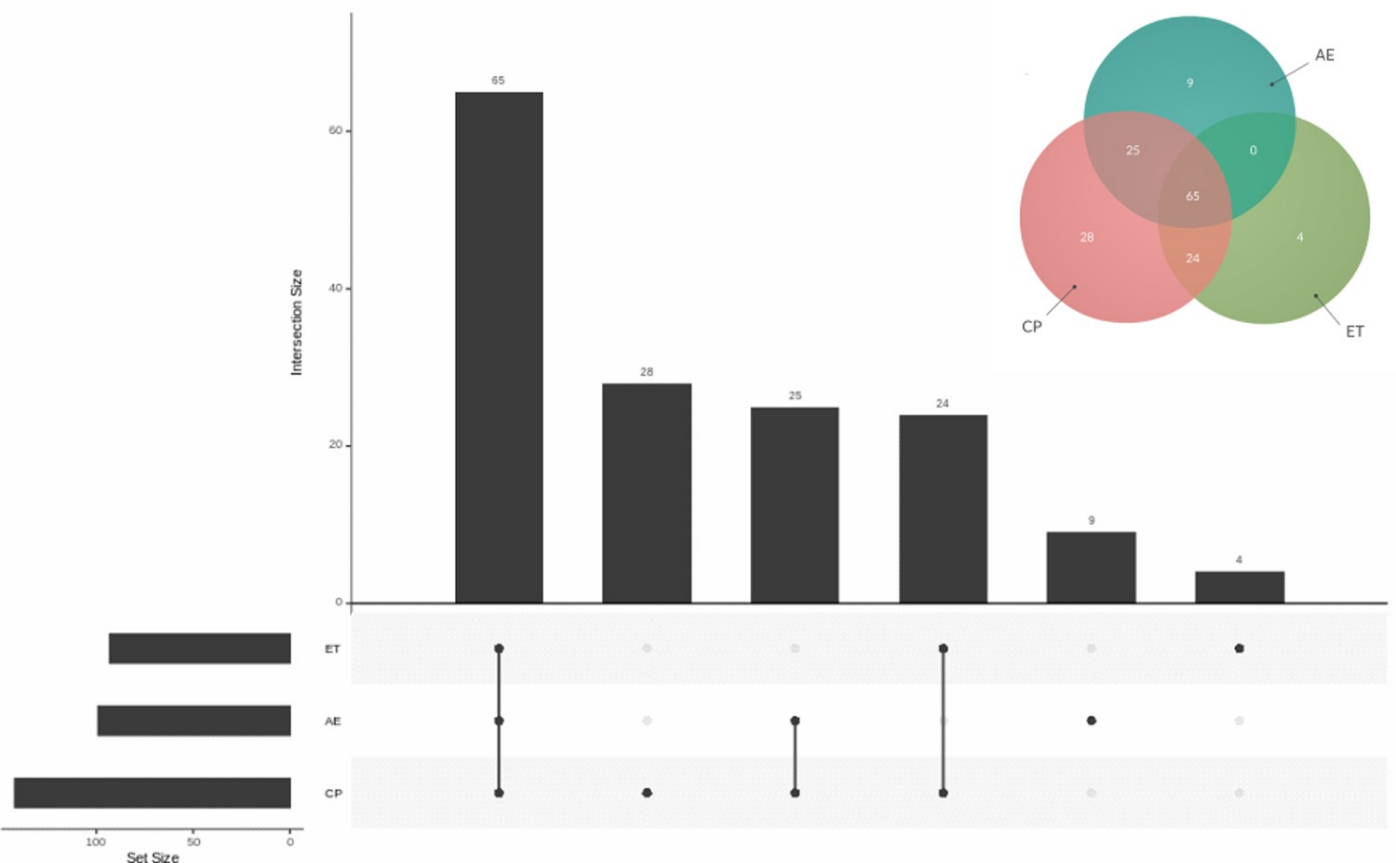
Venn diagram and UpSet plot of the Argentinian samples. Venn diagram and UpSet plot show the number of shared OTUs between chicken cecal Argentinian samples designated as ET: Experimental Trial, CP: Conventional Poultry, and AE: Agroecological Farm.

Chickens under commercial (CP and AE) or experimental conditions (ET) shared a core microbiota composed of 65 classified OTUs (Fig 4), which were assigned to seven phyla and 38 families. Fifteen of them were above 1% at least in one sample, thus they were considered highly abundant. The shared OTUs were dominated by *Bacteroides* genus, Rikenellaceae family, Clostridiales order, and Ruminococcaceae family. All these taxa showed variations in relative abundance among samples (Table 5).

**Table 5:** List of shared OTUs among chicken cecal Argentine samples.

Phylum	Order	Family	Genus	ET	AE	CP
Bacteroidetes	Bacteroidales	Bacteroidaceae	Bacteroides	33.47	18.28	18.56
Bacteroidetes	Bacteroidales	Rikenellaceae	Unclassified	5.86	1.46	15.45
Bacteroidetes	Bacteroidales	[Barnesiellaceae]	Unclassified	5.50	1.27	8.39
Firmicutes	Lactobacillales	Lactobacillaceae	Lactobacillus	2.87	0.24	3.80
Firmicutes	Clostridiales	Unclassified	Unclassified	18.76	7.86	11.97
Firmicutes	Clostridiales	Lachnospiraceae	Unclassified	4.38	0.59	1.92
Firmicutes	Clostridiales	Lachnospiraceae	[Ruminococcus]	2.44	3.24	2.74
Firmicutes	Clostridiales	Ruminococcaceae	Unclassified	10.08	2.21	7.94
Firmicutes	Clostridiales	Ruminococcaceae	Faecalibacterium	0.36	0.77	3.60
Firmicutes	Clostridiales	Ruminococcaceae	*Oscillospira*	2.62	1.63	4.23
Firmicutes	Clostridiales	Ruminococcaceae	*Ruminococcus*	4.37	0.33	2.38
Firmicutes	Clostridiales	Veillonellaceae	*Megamonas*	0	1.12	1.91
Firmicutes	Clostridiales	Veillonellaceae	*Phascolarctobacterium*	2.49	5.77	2.39
Proteobacteria	Burkholderiales	Alcaligenaceae	Sutterella	0.97	2.34	0.64
Proteobacteria	Enterobacteriales	Enterobacteriaceae	Unclassified	1.91	0.84	0.31

The taxonomic classification of the shared OTUs with relative abundance above 1% is shown down to the genus level.

ET: Experimental Trial, CP: Conventional Poultry, AE: Agroecological Farm

## Discussion

As in other vertebrates, the gut microbiota composition of birds is influenced by genetic and non-genetic factors. Understanding the contribution of these factors on the microbial community structure is critical to develop a modulation strategy for improving poultry production. Some studies have found that non-genetic factors are more important in structuring the microbiota than the genetics ones [2]. Likewise, our results showed that the geographic location plays a relevant role in shaping the gut microbiota of chickens than any other evaluated factors. This finding is in accordance with previous works studying GIT microbiota modulation in humans and chicken [5, 8, 9].‬‬‬‬‬‬‬‬‬‬‬‬‬‬‬‬‬‬‬‬‬‬‬‬‬‬‬‬‬‬‬‬‬‬‬‬‬‬‬‬‬‬‬‬‬‬‬‬‬‬‬‬‬‬‬‬‬‬‬‬‬‬‬‬‬‬‬‬‬‬‬‬‬‬‬‬‬‬

Particularly, geographic gradient seems to shape microbiota in young European infant (age 6 weeks) [4]. In this age group, the abundance of Bacteroidaceae, Enterobacteriaceae, and Lactobacillaceae remarkably varied according to the location. Indeed all these families presented higher relative abundance in infants from southern European countries. Our results in European chickens showed a similar pattern, with the highest differences found at extreme latitudes. The relative abundances for Lactobacillacea and Enterobacteriaceae were lower in samples from northern countries (Germany) than in samples from southern countries (Slovenia, Croatia, and Hungary). The opposite geographic gradient pattern came out for Bacteroidetes phylum (Fig 2 and S2 Table).

An analysis of the influence of geography upon microbiota of birds with fermenting crop confirmed that intra-population distances were smaller than between populations, where the differences in the crop microbiota were mostly assigned to environmental differences [6]. In another study, Hird *et al*. [7] suggested that genetics might play less influence in comparison to non-genetic factors like locality, age, and diet in shaping passerine gut microbiota. Finally, in a broader avian study, Waite and Taylor [25] attributed the composition of GIT microbiota mostly to host and location.

One of the main challenges when extracting information from public sequence resources is to take into account the biases and limitations of the methodological approaches that could confound the outcome from the bioinformatics workflow. Sequencing depth indirectly determines the abundance of the bacterial species. Indeed, the detection of rare OTUs requires the presence of many sequences per sample [26]. This constraint can be overcome by performing a normalization data step through random subsampling and total sum-scaling method. Additionally, we considered three factors related to methodological approaches (DNA extraction kit, the variable region of the 16S rRNA, and the sequencing platform) in the analysis. Although Fouhy *et al*. [27] found that the extraction method had a low effect on overall composition, Kennedy *et al*. [28] obtained significant differences in relative abundance associated with different DNA extraction methods. Walker *et al*. [29] and Fouhy *et al*. [27] found the PCR primer sequences are critical determinants of the final bacterial sequences profile. To overcome for potential bias due to both variable target region and primer pairs we used the closed reference OTU picking approach for analyzing the worldwide samples, at the risk of discarding novel real reads. Finally, Allali *et al*. [30] demonstrated significant differences between sequencing platforms and library preparation protocols in the determination of microbial diversity and species richness.

In our work, we corroborated, according to R2 values, that the sequencing platform, extraction kit, and the variable sequenced region indeed have an impact though lesser than the geographic origin (Table 2). Moreover, the results from the two datasets from Argentina (Table 1 and Fig 1) significantly grouped together, yet coming not only from different trials but also obtained in different time-frames.

The Mantel test [31] is a powerful tool for analyzing multivariate data, particularly for data sets expressed through pairwise distances [32]. In this regard, our results showed a significant correlation between beta diversity (unweighted UniFrac) and haversine geographical distances, reinforcing the major influence of geography over any other factor that may arise from methodological constraints.

Others factors, like intestinal portion and age, seemed not impact on microbiota modulation to the same extent as geography. For instance, Malaysia samples clustered together even though they belonged to different sections of GIT (ileum and cecum) and two different age groups (21 and 42 days). On the other hand, the diet, which is one of the most studied parameter in the bibliography, could be a determining influence factor. However, some of the datasets used in our analysis included more than one treatment, but their distribution on PCoA did not show any response to this characteristic. For example Germany dataset include two groups fed with commercial or monocalcium phosphate additive, and the same happened with USA and Argentine samples, which comprised commercial diet versus organic acids, and tannins or bacitracin, respectively (Table 1).

We coined the concept of “local microbiota” because of the highly divergent poultry microbiota linked with geographical location. Resuming the perspective of human-environment geography, local microbiota mirrors the autochthonous non-genetic drivers that modulate bacterial composition. In other words, we propose that geographical location is a convergent feature that conflates non-genetic factors. Thus, local microbiota is worth to take into consideration as the proper base-line for identifying the correlation of the poultry lifestyle and the GIT microbiota, for testing additives on diet to modulate the microbiota as growth promoter factors, and for improving production performance. As an example, the in depth analysis of GIT microbiota of poultry from Argentina allowed us to characterized a native one, bearing Veillonellaceae family in a more predominantly way (> 3%) than in any of the other of the analyzed countries (below 1%) (S2 Table). Particularly, the members of this family are morphologically diverse and obligate Gram-negative anaerobes, capable of degrading organic acids, fermenting lactate, and forming intergeneric coaggregates with other bacteria providing nutrients and protection for all participants [33].

Yet we can talk about an Argentine microbiota, we still can distinguish the existence of specific community structures linked to CF versus ET at the sub local level (Fig 3 and S3 Table). Additionally, diversity indexes from CF are higher than the ones from ET (Observed OTUs, Chao1, Shannon, and Simpson indexes—Table 4). We consider the litter management regimen as the most noticeable feature among the variables that could be responsible of the observed differences between both husbandry practices. The poultry litter consists primarily of a mixture of bedding materials and bird excreta and, unlike in ET, in commercial farms the litter is used throughout the year (from five to six productive cycles). Repeated use of poultry litter, results in considerable changes in the chemical and microbiological conditions of litter [34]. Other authors had also demonstrated, the litter effect on the composition and structure of poultry GIT microbiota. In that sense, our results (Table 4), are in accordance with Wang *et al*. [34] and Cressman *et al*. [35], where the diversity of cecal samples from animals raised in pens with reused litter was significantly greater in comparison to the diversity of those raised using fresh litter.

Clearly differences were observed according to the multidimensional scaling analysis. Fig 3 displays not only the split into experimental or commercial conditions (ET and CF) but also the sub divisions within each group. ET comprises two slightly different experimental designs (ET1 and ET2, Table 1) and CF involves two alternative productive management systems (CP and AE). Notably, although CP encompasses samples from different commercial farms (S1 Table) they are all grouped together, significantly separated from the AE ones.

Despite these differences found into Argentine samples, we described a microbial taxonomic core (Fig 4 and Table 5). The identification of a taxonomic core among Argentine poultry could be useful to evaluate the trends of microbiota dynamics in a more accurate way at regional level. The four more abundant shared OTUs were *Bacteroides*, Rikenellaceae, Clostridiales and Ruminococcacea, typical members of the chicken GIT.

The cecum harbors a bacterial community that allows anaerobic fermentation of cellulose and other substrates [19]; many of the members of this community belong to the Bacteroidetes phylum. Among Bacteroidetes, *Bacteroides* was the most abundant genus in the Argentine core, capable of performing an efficient polysaccharide degradation and producing short-chain volatile fatty acids [36]. On the other hand, the Rikenellaceae family generally indicates gastrointestinal good−health. Members of this family seem to be specialized in the digestive tract of a number of different animals, and have been identified both in fecal and GIT samples [37].

Within Firmicutes phylum, Clostridia class dominated the ileum and cecum microbiota of healthy chickens [14]. Rinttilä and Apajalahti [38] suggest that most members of Clostridia are nonpathogenic, encompassing many beneficial bacteria like cellulose and starch degraders. In accordance, the Clostridiales order was the most abundant member of the Firmicutes phylum in the Argentine core. Among the Clostridia class Ruminococcaceae and Lachnospiraceae were the most abundant families similar to what has been described by Oakley *et al*. [16] and Neumann and Suen [39] for cecum of broiler chicken.

## Conclusion

The results of the present study reinforce the role of geographic location as a native modulator factor of microbial community present in chicken gastrointestinal tract. We believe that a global picture of diversity is emerging, despite the limitations of cross-study meta-analyses due mainly to methodological biases. Therefore, here we report a conservative approach, using closed reference OTU picking due to different 16S gene regions involved, limiting the potential for sequencing noise to interfere with the results at the cost of perhaps discarding real, novel reads.

A larger number of studies should be included in future analyzes to validate the results to a wider extent, to support the similarities in the composition within the same country (or same latitude).

Additionally, this study is the first report describing the Argentine microbiota for experimental and commercial farms that could be considered as first baseline approximation when testing modulators of the GIT microbiota in specific contexts to improve poultry health and production. However, further investigations are required to better link the environmental conditions with microbiota modulation parameters so as to develop novel strategies for improving production outputs.

## Supporting information

S1 Fig Clustering analysis of chicken GIT microbial communities at the genus level, based on Bray-Curtis dissimilarity, from different geographic locations. (TIF)

S2 Fig Relative abundance of bacteria at the family level in the chicken GIT samples evaluated from different geographic locations. Geographic locations were designated as AUS: Australia, ARG: Argentina, CRO: Croatia, GER: Germany, HUN: Hungary, MAL: Malaysia, SLO: Slovenia, and USA: United States. The taxonomic classification is expressed as p_: phylum, c_: class, o_: order, and f_: family.
(TIF)

S1 Table Sampling data from Argentinian poultry commercial farms. (XLSX)

S2 Table Statistical analysis for relative abundance of the predominant families in the chicken GIT samples. Chicken GIT samples were evaluated from different geographic locations designated as AUS: Australia, ARG: Argentina. CRO: Croatia, GER: Germany, HUN: Hungary, MAL: Malaysia, SLO: Slovenia, and USA: United States.
(XLSX)

S3 Table Statistical analysis for predominant families in chicken cecal Argentinian microbiota. Argentinian samples were designated as CP: Conventional Poultry, ET: Experimental Trial, and AE: Agroecological Farm.
(XLSX)

## References

[1] Kohl KD. Diversity and function of the avian gut microbiota. J Comp Physiol B Biochem Syst Environ Physiol. 2012;182(5):591–602. 10.1007/s00360-012-0645-z 22246239

[2] Shang Y, Kumar S, Oakley B, Kim WK. Chicken gut microbiota: Importance and detection technology. Front Vet Sci. 2018;5(OCT). 10.3389/fvets.2018.00254 30406117 PMC6206279

[3] Yang Y, Ashworth AJ, Willett C, Cook K, Upadhyay A, Owens PR, et al Review of Antibiotic Resistance, Ecology, Dissemination, and Mitigation in U.S. Broiler Poultry Systems. Front Microbiol. 2019;10(November):1–10. 31803164 10.3389/fmicb.2019.02639 PMC6872647

[4] Fallani M, Young D, Scott J, Norin E, Amarri S, Adam R, et al Intestinal Microbiota of 6-week-old Infants Across Europe: Geographic Influence Beyond Delivery Mode, Breast-feeding, and Antibiotics. J Pediatr Gastroenterol Nutr. 2010;51(1):77–84. 10.1097/MPG.0b013e3181d1b11e 20479681

[5] Yatsunenko T, Rey FE, Manary MJ, Trehan I, Dominguez-Bello MG, Contreras M, et al Human gut microbiome viewed across age and geography. Nature. 2012;486:222–8. 10.1038/nature11053 22699611 PMC3376388

[6] Godoy-Vitorino F, Leal SJ, Díaz WA, Rosales J, Goldfarb KC, García-Amado MA, et al Differences in crop bacterial community structure between hoatzins from different geographical locations. Res Microbiol. 2012;163:211–20. 10.1016/j.resmic.2012.01.001 22313738

[7] Hird SM, Carstens BC, Cardiff SW, Dittmann DL, Brumfield RT. Sampling locality is more detectable than taxonomy or ecology in the gut microbiota of the brood-parasitic Brown-headed Cowbird (Molothrus ater). PeerJ. 2014;2:e321 10.7717/peerj.321 24711971 PMC3970801

[8] Videnska P, Rahman MM, Faldynova M, Babak V, Matulova ME, Prukner-Radovcic E, et al Characterization of egg laying hen and broiler fecal microbiota in poultry farms in Croatia, Czech Republic, Hungary and Slovenia. PLoS One. 2014;9(10):e110076 10.1371/journal.pone.0110076 25329397 PMC4199679

[9] Zhou X, Jiang X, Yang C, Ma B, Lei C, Xu C, et al Cecal microbiota of Tibetan Chickens from five geographic regions were determined by 16S rRNA sequencing. Microbiologyopen. 2016;5(5):753–62. 10.1002/mbo3.367 27139888 PMC5061713

[10] Turner BL. Contested identities: Human-environment geography and disciplinary implications in a restructuring academy. Ann Assoc Am Geogr. 2002;92(1):52–74.

[11] Siso Quintero GJ. ¿Qué es la geografía? Terra [Internet]. 2010;26(39):147–82. Available from: https://www.redalyc.org/pdf/589/58922904003.pdf

[12] Kuczynski J, Stombaugh J, Walters WA, González A, Caporaso JG, Knight R. Using QIIME to analyze 16s rRNA gene sequences from microbial communities. Curr Protoc Bioinforma. 2012;Chapter 10:1–20. 10.1002/9780471729259.mc01e05s27 22161565 PMC3249058

[13] Witzig M, Da Silva AC, Gree-Engert R, Hoelzle K, Zeller E, Seifert J, et al Spatial variation of the gut microbiota in broiler chickens as affected by dietary available phosphorus and assessed by T-RFLP analysis and 454 pyrosequencing. PLoS One. 2015;10(11). 10.1371/journal.pone.0143442 PMC4654470 26588075

[14] Mohd Shaufi MA, Sieo CC, Chong CW, Gan HM, Ho YW. Deciphering chicken gut microbial dynamics based on high-throughput 16S rRNA metagenomics analyses. Gut Pathog [Internet]. 2015;7(4). Available from: http://www.pubmedcentral.nih.gov/articlerender.fcgi?artid=4372169&tool=pmcentrez&rendertype=abstract 10.1186/s13099-015-0051-7 25806087 PMC4372169

[15] Griffiths RI, Whiteley AS, O’Donnell AG, Bailey MJ. Rapid method for coextraction of DNA and RNA from natural environments for analysis of ribosomal DNA- and rRNA-based microbial community composition. Appl Environ Microbiol. 2000;66(12):5488–91. 10.1128/aem.66.12.5488-5491.2000 11097934 PMC92488

[16] Oakley BB, Buhr RJ, Ritz CW, Kiepper BH, Berrang ME, Seal BS, et al Successional changes in the chicken cecal microbiome during 42 days of growth are independent of organic acid feed additives. BMC Vet Res [Internet]. 2014;10(282):282 Available from: http://www.pubmedcentral.nih.gov/articlerender.fcgi?artid=4251860&tool=pmcentrez&rendertype=abstract 10.1186/s12917-014-0282-8 25427406 PMC4251860

[17] Yu Z, Morrison M. Improved extraction of PCR-quality community DNA from digesta and fecal samples. Biotechniques. 2004;36(5):808–12. 10.2144/04365ST04 15152600

[18] Stanley D, Geier MS, Chen H, Hughes RJ, Moore RJ. Comparison of fecal and cecal microbiotas reveals qualitative similarities but quantitative differences. BMC Microbiol [Internet]. 2015;15(51). Available from: http://www.ncbi.nlm.nih.gov/pmc/articles/PMC4403768/ 10.1186/s12866-015-0388-6 25887695 PMC4403768

[19] Díaz Carrasco JM, Redondo EA, Pin Viso ND, Redondo LM, Farber MD, Fernández Miyakawa ME. Tannins and Bacitracin Differentially Modulate Gut Microbiota of Broiler Chickens. Biomed Res Int. 2018;2018:11 10.1155/2018/1879168 29682522 PMC5841071

[20] Edgar RC, Haas BJ, Clemente JC, Quince C, Knight R. UCHIME improves sensitivity and speed of chimera detection. Bioinformatics. 2011; 10.1093/bioinformatics/btr381 21700674 PMC3150044

[21] Edgar RC. Search and clustering orders of magnitude faster than BLAST. Bioinformatics. 2010;26(19):2460–1. 10.1093/bioinformatics/btq461 20709691

[22] Oksanen J, Blanchet FG, Friendly M, Kindt R, Legendre P, McGlinn D, et al Package “vegan”. Community Ecology Package. 2017; Available from: https://github.com/vegandevs/vegan/issues

[23] Lozupone C, Knight R. UniFrac: a new phylogenetic method for comparing microbial communities. Appl Environ Microbiol. 2005;71(12):8228–35. 10.1128/AEM.71.12.8228-8235.2005 16332807 PMC1317376

[24] Parks DH, Tyson GW, Hugenholtz P, Beiko RG. STAMP: Statistical analysis of taxonomic and functional profiles. Bioinformatics. 2014;30(21):3123–4. 10.1093/bioinformatics/btu494 25061070 PMC4609014

[25] Waite DW, Taylor MW. Characterizing the avian gut microbiota: Membership, driving influences, and potential function. Front Microbiol. 2014;5:223 10.3389/fmicb.2014.00223 24904538 PMC4032936

[26] Weiss S, Xu ZZ, Peddada S, Amir A, Bittinger K, Gonzalez A, et al Normalization and microbial differential abundance strategies depend upon data characteristics. Microbiome [Internet]. 2017;5(27):1–18. Available from: http://microbiomejournal.biomedcentral.com/articles/10.1186/s40168-017-0237-y 10.1186/s40168-017-0237-y 28253908 PMC5335496

[27] Fouhy F, Clooney AG, Stanton C, Claesson MJ, Cotter PD. 16S rRNA gene sequencing of mock microbial populations-impact of DNA extraction method, primer choice and sequencing platform. BMC Microbiol. 2016;16(123):13 10.1186/s12866-016-0738-z 27342980 PMC4921037

[28] Kennedy NA, Walker AW, Berry SH, Duncan SH, Farquarson FM, Louis P, et al The impact of different DNA extraction kits and laboratories upon the assessment of human gut microbiota composition by 16S rRNA gene sequencing. PLoS One. 2014;9(2):e88982 10.1371/journal.pone.0088982 24586470 PMC3933346

[29] Walker AW, Martin JC, Scott P, Parkhill J, Flint HJ, Scott KP. 16S rRNA gene-based profiling of the human infant gut microbiota is strongly influenced by sample processing and PCR primer choice. Microbiome [Internet]. 2015;1–11. Available from: 10.1186/s40168-015-0087-4 26120470 PMC4482049

[30] Allali I, Arnold JW, Roach J, Cadenas MB, Butz N, Hassan HM, et al A comparison of sequencing platforms and bioinformatics pipelines for compositional analysis of the gut microbiome. BMC Microbiol [Internet]. 2017;17(194):1–16. Available from: http://bmcmicrobiol.biomedcentral.com/articles/10.1186/s12866-017-1101-8 10.1186/s12866-017-1101-8 28903732 PMC5598039

[31] Mantel N. The detection of disease clustering and a generealized regression approach. Cancer Res. 1967;27:209–20. 6018555

[32] Legendre P, Fortin MJ, Borcard D. Should the Mantel test be used in spatial analysis? Methods Ecol Evol. 2015;6(11):1239–47.

[33] Gronow S, Welnitz S, Lapidus A, Nolan M, Ivanova N, Del Rio TG, et al Complete genome sequence of Veillonella parvula type strain (Te3T). Stand Genomic Sci. 2009;1:57–65. 10.4056/sigs.521107 PMC3035260 21304678

[34] Wang L, Lilburn M, Yu Z. Intestinal Microbiota of Broiler Chickens As Affected by Litter Management Regimens. Front Microbiol. 2016;7:593 10.3389/fmicb.2016.00593 27242676 PMC4870231

[35] Cressman MD, Yu Z, Nelson MC, Moeller SJ, Lilburn MS, Zerby HN. Interrelations between the microbiotas in the litter and in the intestines of commercial broiler chickens. Appl Environ Microbiol. 2010;76(19). 10.1128/AEM.00180-10 20693454 PMC2950482

[36] Saxena S, Saxena VK, Tomar S, Sapcota D, Gonmei G. Characterization of Caecum and Crop Microbiota of Indian Indigenous Chicken Targeting Multiple Hypervariable Regions within 16S rRNA gene. Br Poult Sci. 2016;57(3):381–9. 10.1080/00071668.2016.1161728 26962896

[37] Graf J. The Family Rikenellaceae. In: Eugene R., Stephen D.E.F.L., Erko TF S., editor. The Prokaryotes: Other Major Lineages of Bacteria and The Archaea. Springer-Verlag, Berlin Heidelberg; 2014 p. 857–859.

[38] Rinttilä T, Apajalahti J. Intestinal microbiota and metabolites—Implications for broiler chicken health and performance. J Appl Poult Res. 2013;22(3):647–58.

[39] Neumann AP, Suen G. Differences in major bacterial populations in the intestines of mature broilers after feeding virginiamycin or bacitracin methylene disalicylate. J Appl Microbiol. 2015; 10.1111/jam.12960 26425940

