# Supplementary figures and images for: Geography as non-genetic modulation factor of chicken cecal microbiota
Source: PLoS One. 2021 Jan 6;16(1):e0244724. doi: 10.1371/journal.pone.0244724 (PMC7787451; doi:10.1371/journal.pone.0244724)

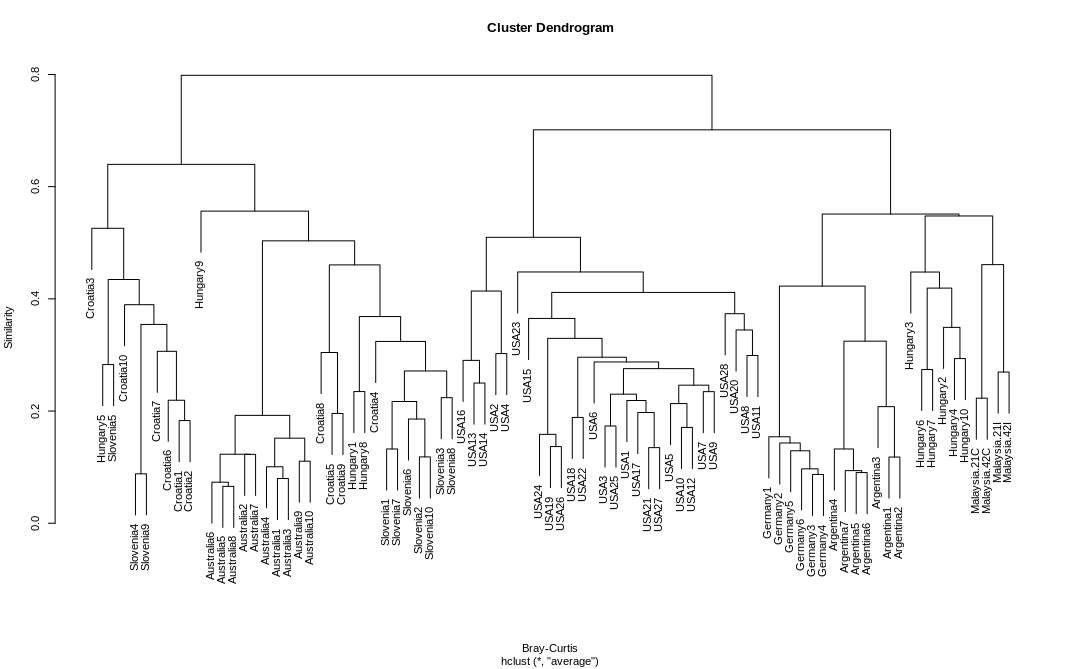

Supplement: S1 Fig — (TIF) [file pone.0244724.s001.tif]

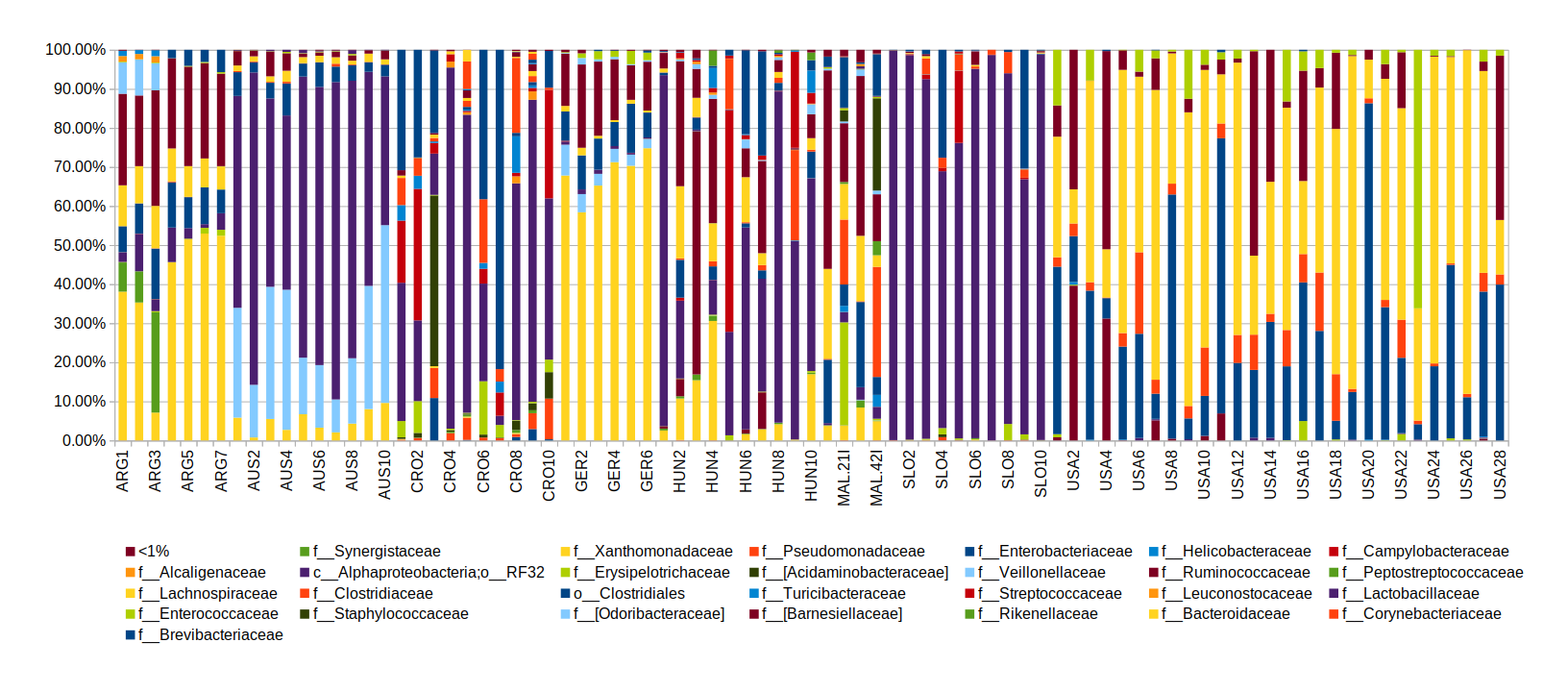

Supplement: S2 Fig — Geographic locations were designated as AUS: Australia, ARG: Argentina, CRO: Croatia, GER: Germany, HUN: Hungary, MAL: Malaysia, SLO: Slovenia, and USA: United States. The taxonomic classification is expressed as p_: phylum, c_: class, o_: order, and f_: family. (TIF) [file pone.0244724.s002.tif]
