# Supplementary material for: Geography as non-genetic modulation factor of chicken cecal microbiota
Source: PLoS One. 2021 Jan 6;16(1):e0244724. doi: 10.1371/journal.pone.0244724 (PMC7787451; doi:10.1371/journal.pone.0244724)
Supplement: Attachment — Submitted filename: Response_to_Reviewers.docx [file pone.0244724.s006.docx]

PONE-D-20-04279
Geography as non-genetic modulation factor of chicken cecal microbiota

We are sending a revised version of our manuscript. We are grateful to the Editor and the Reviewers for the attentive reading of our manuscript and helpful insight. The changes suggested by the Reviewers have contributed substantially to improving the manuscript. We carefully evaluated the suggestions, revised the text and hope the manuscript is acceptable for the publication in the present format.

Our responses (in italics) to Reviewers' comments:

Review Comments to the Author

Reviewer #1: In this article authors performed analysis based on 16S rRNA gene amplicon sequences from cecum/fecal/ileum broiler samples available in public databases. They compared experimental trial (ET) broilers from different geographical locations. Additionally, they included Argentina broiler farms samples taken and processed by them. In this study different geographical locations are defined as different countries. Each country included only one study. They concluded that geographical location played a major influence in shaping chicken gut microbiome over any other evaluated factor based on R2 (Adonis). Authors indicated that the sequencing platform, extraction kit and the variable sequenced region have a lower impact than the geographic origin, based on R2 values.

Major comments

I consider that the general hypothesis of this article cannot be tested using the methodology chosen by the authors. Therefore, it would not be appropriate to conclude that geography is the factor that most influences bacterial composition.

Table 2 shows the R2 of each of eight fit factors that were significant (Adonis). These included geographic location, sequenced region, diet, extraction kit, intestinal portion, age, genetic line and platform. The greatest adjustment obtained was by geographic location. However, the variables being evaluated are correlated within geographic location and this effect should be controlled. R2 inflated value may be warning an overfitting of the model. It would not be appropriate to suggest that geographic location other than differences between studies, which should be considered as the combination of multiple methodological variables, is having an effect. Thus, the closeness of samples from Slovenia, Croatia and Hungary in PCoA from figure 1 may be due to the fact that they came from the same study. Moreover, Germany and the USA, that are countries in the same latitude as Slovenia, Croatia and Hungary, were separated, and they came from different studies.

The definition of a “local microbiota” between countries should only be defined as such when at least more than one study per country or per locality is carried out. Furthermore, these studies must have a certain degree of standardization of sample processing and sequencing protocols as previous studies (1,2,3,4).

References
1. Godoy-Vitorino F, Leal SJ, Díaz WA, Rosales J, Goldfarb KC, 429 García-Amado MA, et al. Differences in crop bacterial community structure between hoatzins from different geographical locations. Res Microbiol. 2012;163:211–20.

2. Hird SM, Carstens BC, Cardiff SW, Dittmann DL, Brumfield RT. Sampling locality is more detectable than taxonomy or ecology in the gut microbiota of the brood-parasitic Brown headed Cowbird (Molothrus ater). PeerJ. 2014;2:e321.

3. Videnska P, Rahman MM, Faldynova M, Babak V, Matulova ME, Prukner-Radovcic E, et al. Characterization of egg laying hen and broiler fecal microbiota in poultry farms in Croatia, Czech Republic, Hungary and Slovenia. PLoS One. 2014;9(10):e110076.

4. Zhou X, Jiang X, Yang C, Ma B, Lei C, Xu C, et al. Cecal microbiota of Tibetan Chickens from five geographic regions were determined by 16S rRNA sequencing.

*We agree with the Reviewer that we ought to control for any over-fitting of the model. However, given the scarcity of more than one data set per country we alternatively selected pairs of experiments with the same state for any variable, so as to support the comparison. That is, the data set from USA and Australia target the 16S V1-V3 region, whereas V3-V4 region was the one used in the data sets from Eastern Europe and Argentina. Furthermore, Illumina was the sequencing platform used in the experiments from Argentina and Malaysia, and Roche for Eastern Europe, Germany and the USA ones. Additionally, we performed the Mantel test obtaining a significant correlation between distances matrix from geographic and microbial composition.*

*Since public datasets were used, the sampling and sequencing process cannot be standardized ex-post. However, we still think that valid information could be deduced from cumulative data available in public resources. For stripping out the methodology effect we analyzed as much variables as possible, such as the DNA extraction kit, the sequencing primers or* *amplified region, and the sequencing platform. Although they exerted significant effect, their influence was lesser than the one coming from the location (table 2). Finally, we coined the “local microbiota” definition after testing the location hypothesis using Argentinian data coming from different trials and time-frames. For this analysis we fulfilled the suggestion of using at least more than one set per locality.*

Reviewer #2: The manuscript presented by Pin Viso and collaborators presents a meta-analysis of broilers' cecal microbiota from multiple countries and self-generated data about Argentinian poultry cecal microbiota. The analysis and the data presented are well processed, using standard pipelines. However, you did not declare precise run parameters, and I suggest to writhe the whole bioinformatics pipeline as a piece of supplementary information uploaded to figshare or Github. I would encourage the authors to seek alternative explanations beyond geography for microbiome structuring. The declared diets for all the other studies are broilers' standardized commercial diets (SDC), with slight modifications in each country. Previous works have shown that tiny changes in diet could alter the microbiome output in chickens like a recent publication adding galactooligosaccharides to chicken diet (<https://msystems.asm.org/content/5/1/e00827-19>). Another possible explanation could be poultry management in each location. Adonis results are suggestive of supporting significant geographical effects further. I would recommend using Mantel tests to correlate geographical distances among locations and the microbiome compositions. Some of your conclusions have no data back, and you could move them to the discussion. Another possibility is the sequence length of each study since you are combining multiple sequencing platforms with different efficiency and output. Please deposit OTU tables, taxonomic assignments, representative sequences in a repository (figshare) to share among other people working in bird microbiomes, and it would facilitate further meta-analysis

*We appreciate the suggestions regarding seeking for alternative explanations. Although we sustained the geographical location as the driver we went deeper into the subject of Geography definitions for gaining clarity. To that end, we supported our argument using specific bibliography (Turner 2002, Siso Quintero 2010) and recalling the human-environment theoretical position, we proposed that geographical location was a convergent feature that conflated non-genetic factors (added in “Introduction” L75-81and “Discussion” L359-363.).*

*On the other hand, we acknowledge the suggestions of using Mantel test to support the results (L181-183). In addition,* *QIIME scripts together with intermediate results can be reach trough figshare: https://doi.org/*[*10.6084/m9.figshare.c.4993856*](https://doi.org/10.6084/m9.figshare.c.4993856)

Particular comments:
L29 Missing comma "genotype, or geographical"

*Correction has been done*

L93-94 Table 1. Please upload your data to NCBI/EBI/DDBJ (AVAILABLE UNDE REQUEST). Add sequence length average of each platform, and the total output in base pairs for each compared project.

*All sequence data generated for this paper was deposited in the NCBI Sequence Read Archive database under the BioProject accession number PRJNA579062. Additionally, QIIME scripts and intermediate results are available from figshare (*[*https://doi.org/10.6084/m9.figshare.c.4993856*](https://doi.org/10.6084/m9.figshare.c.4993856)*).*

*Sequenced cited in Table 1 were obtained after requesting to J. Diaz Carrasco (Diaz Carrasco et al. 2018 and Diaz Carrasco unpublished).*

*We consider unnecessary to add sequence length average of each platform in Table 1, as long as the resulting files, after merging Forward (F) and Reverse (R) ones, turn out to be the 16S amplified region which range of variation is already considered in Table 1 under “Region sequenced”. Similarly, data is subjected to normalization before composition and alpha and beta diversity analysis, so total output won’t exert any influence.*

L118-119 Add details about PCR experiments run conditions, primers used, etc.

*Thank you for bringing up to this matter. Not only sequencing of the samples but also amplification were performed at Macrogen Inc,(we reworded that paragraph). Nucleotide primer sequences were added in L131--132.*

L128-145 Description is neat, however for using reproducibility, please prepare a notebook as supplementary information; you could use the aid of jupyter-notebooks or merely a text with all your bioinformatics and statistical procedures cut-offs.

*Added in L153-154. QIIME scripts and intermediate results are available from figshare (*[*https://doi.org/10.6084/m9.figshare.c.4993856*](https://doi.org/10.6084/m9.figshare.c.4993856)*)*

L150 What is the alpha cut-off value of STAMP?

*Added in L171*

Table 2 Please include average sequence length and sequencing output for the multiple datasets in your analysis using ADONIS.

*Answered above, in relation to Table 1 suggestions.*

L185 Missing comma, check out the use of commas with lists in English: <https://www.grammarly.com/blog/comma/> not quite the same as in Spanish with the last conjunction.
*Corrections have been done all along the text.*

L208 Rewrite from "under ET" to "under experimental trail (ET)"
*Modification has been done (L223)*

L214 How many OTUs did you find in all the compared samples? The overall alpha diversity metrics.

*Added (L228).*

Since space is no limitation in PLoS one, I would recommend you upload Supplemental Figure 1 to the main text to have the microbial structure description of your comparisons.
*We decline this suggestion, we consider Fig 2 depicts the microbial structure satisfactorily. It is our understanding that Supp Fig 1 would not add fundamental information that worth to include in the main text.*

Table 4 there are some inconsistencies between the Shannon values reported here and the ones reported in Table 3 for Argentina, please clarify.

*The data set analyzed in Table 4 is rather different from the one analyzed in Table 3. For the Characterization of Argentine cecal microbiota (Table 4) we consider not only the Experimental Treatment data used in the among countries comparison (ARG_ET 1 & ARG_ET2, Table 1), but also 17 extra samples that came from the same data set but were from a different animal category (wider range of ages). Reworded in L226.*

L285 Rewrite "more prevelailing" to "relevant"
*Modification has been done (L302)*

L288 "infant", please describe human infants.
*Infant category corresponded to 6 weeks of age (Added in L307)*

L289 Missing comma "Enterobacteriaceae, and"
*Done*

L310 Define TSS method
*Added in L329, described in MyM L145-146*

L326 Discuss poultry management, diet, etc.
*Factors such as intestinal portion, age, and diet are discussed from L349 to L359. Additionally, we added more precise information in the paragraph corresponding to “local microbiota” definition (L360-364).*

L333 Define what were the experimental treatments in the other works
*Treatments are defined in Table 1 (Diet column) and all along the paragraph from L349 to L359*

L338 "local microbiota" to endemic?
*It is our understanding that endemic species are found only in the area under consideration and nowhere else. GIT is a rather stable environment in itself, and the variation among microbiota is mainly due to changes in the relative abundance. Our definition of “local microbiota” is in the framework of human-environment geography, being location an idealized geographical space*.

L343 and/or Please choose one
*Modification has been done (L 367)*

L374 Describing a microbial taxonomic core is always possible it's just set theory. So rewrite to "we described a microbial taxonomic core"
*Modification has been done ( L 397-398)*

L374 I recommend you to try Upset to calculate your core and even compare with the other described works. <https://cran.r-project.org/web/packages/UpSetR/vignettes/basic.usage.html>
*We acknowledge the suggestion. We modified the figure 4 for improving further interpretations. We are uploading it as “new_Fig4”*

L374-375 Speculation, what is the basis for stability or homeostasis. How do you measure it?
*We agree is mere speculation, being out of the reach of our results at this stage. We deleted the phrase L398-399*

L377 remove prefixes g__ f__ c__
*Done L402*

L389 ellaborate the "All existing evidence"
*We added the reference of Rinttilä and Apajalahti, a revision work that thoroughly described microbiota implications for broiler chickens health L413*

L400-402 Move this to the discussion, where you can elaborate on your idea. However, it is outside the scope of your manuscript. "This local microbiota could improve the understanding of the poultry lifestyle on production performance"
*We agree is just speculation, we deleted the phrase in accordance to the suggestion of toning down the conclusions. L425-426*

L404 modify to "first baseline approximation"
*Done ( L427-428)*

L405 rewrite "researches" to "investigations"

*Done ( L430)*
