# Supplementary material for: Geography as non-genetic modulation factor of chicken cecal microbiota
Source: PLoS One. 2021 Jan 6;16(1):e0244724. doi: 10.1371/journal.pone.0244724 (PMC7787451; doi:10.1371/journal.pone.0244724)
Supplement: Attachment — Submitted filename: Response_to_Reviewers.docx [file pone.0244724.s007.docx]

October 2020

Luis David Alcaraz, Ph.D.
Academic Editor
PLOS ONE

Dear Editor,

We are sending a revised version of the manuscript entitled “Geography as non-genetic modulation factor of chicken cecal microbiota” by Pin Viso *et al.*, to be considered for publication in PLOS ONE.

We are grateful to the Reviewers for the attentive and careful reading of our manuscript and helpful insight. The changes suggested by the Reviewers have contributed substantially to improve the manuscript. We evaluated in detail the suggestions, revised the text, and reanalyzed part of the data in the frame of a more conservative approach. We hope the manuscript is acceptable for the publication in the present format.

On the following pages, you will find our answer (in red) to all the reviewers’ comments and suggestions.

Sincerely yours,

Dr. Marisa Farber

Review Comments to the Author

Reviewer #1: I am concerned about the over fitting the authors can not control due to the scarcity of more than one data set per country, particularly important because the whole article is focused in a conclusion that cannot be well supported. Mantel analysis including geographical distance may be also bias do to the presence of a single study which includes 3 different countries that are geographically close to each other. I consider it essential to detect similarities in composition among different studies from the same country (or same latitude) to suggest that geography, more than studies, is the main compositional shaping variable.

As we mentioned in the first revision, we agree with the Reviewers that we cannot totally control the over-fitting of the model. In an effort to track for any trend in a more stringent manner, we compared pairs of experiments with the same state for any variable, so as to support the comparison (at least two data sets targeting the same 16S region, at least two that had been used the same sequencing platform, etc.). Additionally, we used two data sets from Argentina coming from different trials and time-frames.

We re-analyzed all data set from geographical location analysis, using a more conservative close reference approach (Line 138-141 M&M), obtaining equivalent results, revealing the geographical trend that prompted our work (Results: Fig1, Table 2, Fig, 2, Table 3, Fig S2, Table S2).

We have rewritten the conclusion section to account for inherent limitations of this kind of studies.

Reviewer #3: The authors present a meta-analysis of cecum/ileum and fecal broiler microbiota based on 16S rRNA gene amplicon sequences taken from public databases including studies in different countries and compared them with their experimental data from broiler samples with different management practices in Argentina. Their conclusions reinforce notions of the geographic location as driver of microbial community composition since other tested factors are not informative enough. Data analysis is sufficiently well done using standard pipelines. Reviewers statistical and technical inquiries have been addressed, as well as the elimination of non supported conclusions.
Detailed descriptions of bioinformatic methods and other inquiries made by Reviewer #2 cannot be consulted. The figshare link is not available at <https://doi.org/10.6084/m9.figshare.c.4993856>

We have made publicly available the additional information under the link: <https://doi.org/10.6084/m9.figshare.c.4993856.v1>

Particular comments:

L204 . Lactoballaceae rewrite Lactobacillaceae

Has been corrected in L 196

L366 Rewrite non-genetic
Has been corrected in L 366

L369 Rewrite local microbiota is worth
Has been corrected in L 369

L372 Rewrite in depth
Has been corrected in L 372

L419 Rewrite plural for degrader

Has been corrected in L 418

Reviewer #4: I found that this is a second round review and the authors already responded to the the previous reviewers comments. I agree with all concerns raised by the previous reviewers. The authors performed the study and analyzed data carefully considering many potential pitfalls that are inherent to this type of meta-analysis. The authors attempted to address all the critiques of the previous reviewers. Nonetheless, I think the validity of the conclusion in this study that geography is a major variable for chicken gut microbiota cannot be supported fully with no room for further discussion. Therefore, the conclusion of this study still has limitations, yet the data presented here has certain value to the research community. I came to the conclusion to support the publication of this manuscript under the following conditions.

1. The authors add a paragraph to present inherent limitations of this study and potential issues associated with the conclusion of the study (as well pointed out by the reviewers 1 and 2 from the 1st round review).

We have rewritten and toned down the conclusion accordingly with the suggestion.

2. I ask the authors to add the information on the PCR primers used in different studies (probably in a supplementary table). Even though two different studies used the same V3-V4 region (for example) they could have used different primer designs (primer length, precise targeting region, degeneracy etc.). Since the variation in the primer design can bring significant changes in the resulting 16S rRNA gene profiles, it is necessary to include the information.

In an recent work (Mancabelli L et al., Microorganisms 2020, 8, 131; doi:10.3390/microorganisms801013), the authors designed a study in order to identify possible correlations between bacterial amplification capabilities and the PCR
primer pairs. To that end, they considered different combinations of primers per each variable region (for example 6 primer pairs targeting the V3-V4), demonstrating that the detected community structure strictly depend on the
particular hypervariable region that was targeted for amplification. Nevertheless, we re-analyzed all geographical data using close reference OTUs picking, as a more conservative approach We have rewritten M&M, Results and discussion session, reporting the new values that account for equivalent geographical trend depicted in the previous version.
